# Complications encountered during Forsus Fatigue Resistant Device therapy

**DOI:** 10.1590/2177-6709.25.3.065-072.oar

**Published:** 2020

**Authors:** Sherif A. Elkordy, Mona M. Salah Fayed, Khaled H. Attia, Amr M. Abouelezz

**Affiliations:** 1 Cairo University, Faculty of Dentistry, Department of Orthodontics and Dentofacial Orthopedics (Cairo, Egypt).; 2 University of Malaya, Faculty of Dentistry, Department of Pediatric Dentistry and Orthodontics (Kuala Lumpur, Malaysia).

**Keywords:** Class II malocclusion, Forsus, Complication, Fixed functional appliance

## Abstract

**Introduction:**

Fixed functional appliances are non-compliant solutions to Class II malocclusion treatment. The clinician, however, should be careful of unexpected complications during the therapy.

**Methods:**

58 female adolescents who presented with Class II malocclusion due to deficient mandible were treated with Forsus Fatigue Resistant Device (FFRD) therapy until an overcorrection to an edge to edge incisor relationship was achieved.

**Results:**

Incisor relationship and overjet were corrected successfully in all the subjects. Twenty-two patients had a complications-free treatment, while several complications were encountered with the remaining 36 subjects. In particular, mandibular canine rotation and development of posterior crossbites were the most common complications, with percentages of 51.7% and 25.9% respectively. Other complications included the breakage and shearing of the extraoral tubes of the first molar bands, and excessive intrusion of the upper first molars.

**Conclusions:**

FFRD is an efficient appliance for treatment of Class II malocclusion; however, different complications were encountered during the appliance therapy. A focus on taking precautions and applying preventive measures can help to avoid such problems, reducing the number of emergency appointments and enhancing the treatment experience with the appliance.

## RESUMO

**Introdução:**

Os aparelhos funcionais fixos são uma solução para o tratamento da má oclusão de Classe II em pacientes não colaboradores. Porém, o ortodontista deve estar ciente das complicações inesperadas decorrentes do seu uso.

**Métodos:**

Cinquenta e oito pacientes adolescentes do sexo feminino com má oclusão de Classe II por deficiência mandibular foram tratadas com o aparelho Forsus FRD até se alcançar uma sobrecorreção, com relação de topo a topo dos incisivos.

**Resultados:**

A relação entre os incisivos e a sobressaliência foram corrigidas com sucesso em todas as pacientes. Vinte e duas pacientes não apresentaram complicações durante o tratamento, enquanto as demais trinta e seis pacientes apresentaram diferentes complicações. As complicações mais comuns foram a rotação do canino inferior e o desenvolvimento de mordida cruzada posterior, com prevalência de 51,7% e 25,9%, respectivamente. As demais complicações incluíram quebra e cisalhamento dos tubos extrabucais das bandas dos primeiros molares, e intrusão excessiva dos primeiros molares superiores.

**Conclusões:**

O FRD é um aparelho eficiente para o tratamento da má oclusão de Classe II. No entanto, diferentes complicações foram encontradas durante o uso desse aparelho. O foco na tomada de precauções e em medidas preventivas pode ajudar a evitar tais complicações, reduzindo o número de consultas de emergência e melhorando a experiência do paciente no tratamento com esse aparelho.

## INTRODUCTION

The benefits of orthodontic treatment include improvement in dental health, function, appearance, and self-esteem. Success of orthodontic treatment is affected by the discomfort caused by the appliances used. It is well documented that such discomfort might reduce the patients’ compliance and satisfaction with the treatment.^1^ Orthodontic appliances can cause unwanted complications, the presence of which may interfere with the treatment quality.^2^ It is thus important for the clinicians to be aware of these potential complications before the start of orthodontic therapy.

Fixed functional appliances were introduced as compliance-free options for treatment of Class II malocclusion. The Forsus Fatigue Resistant Device^3^ (FFRD) (3M Unitek, Monrovia, Calif, USA) was introduced after the earlier Forsus Flat Spring,^4^ and was reported to be successful in the treatment of Class II malocclusion^5^ ^-^ ^7^ and well accepted by the patients.^8^ ^-^ ^11^ A recent systematic review investigated the prevalence of complications with fixed functional appliances, and stated that the incidence of complications is relatively high.^12^ Studying the complications induced by orthodontic appliances has two main aspects: the clinician’s observations and the patients’ responses to assessment questionnaires. Regarding fixed functional appliances, the clinical perspective was previously reported with the Herbst appliance.^13^ ^-^ ^16^ Previous studies that investigated the complications of FFRD were based on patient reporting and acceptance questionnaires.^8^ ^-^ ^11^ ^,^ ^17^

The present clinical report highlights several complications that were encountered during the FFRD therapy in adolescents with Class II malocclusion, and discusses how they were managed. These complications do not underestimate the efficiency nor the acceptability of the appliance. According to Benjamin Franklin axiom that “an ounce of prevention is worth a pound of cure”, knowledge of such complications would be beneficial to the clinicians to take their safety measures during treatment.

## MATERIAL AND METHODS

Fifty-eight adolescent females were consecutively treated, by the same clinician, with FFRD in the outpatient clinic of the Orthodontic Department, Faculty of Dentistry, Cairo University, in the period between 2013 and 2017. The clinician has been using FFRD for three years before this date. The patients’ characteristics were:

» 11-13 years of age.
» Skeletal maturational stage was selected to be stage 3 or 4 from the cervical maturational index according to Baccetti et al.^18^
» Skeletal Class II malocclusion (ANB > 4°, as determined from pre-treatment cephalograms analysis).
» Deficient mandible (SNB < 76°, as reported from pre-treatment cephalograms).
» Class II canines’ relationship and overjet ≥ 5 mm, as measured from pre-treatment study models.

Brackets with 0.022” slot (3M, MBT prescription) were bonded to maxillary and mandibular arches, and a transpalatal arch (TPA) was soldered to orthodontic bands that were cemented to the permanent maxillary first molars. TPA placement intended to control the molar rotation that might be induced by the distally applied force generated by the FFRD on the first molars.^19^ The second permanent molars were not fully erupted in most of the patients at the start of treatment, and thus were not included in the initial leveling stage. Leveling and alignment proceeded until reaching 0.019 x 0.025-in stainless steel archwires, which were cinched distal to the first molars. The mandibular anterior teeth, canine to canine, were tied together with stainless steel ligatures. The mandibular canines were separately ligated with metal ligatures, for additional engagement during the FFRD stage.

The FFRD that was used in this report was the EZ2 module type. The proper size of the FFRD was selected according to the manufacturer instructions. The EZ2 modules of the springs were inserted in the extraoral tubes of the first molars and the push rods of the appliance were inserted distal to the mandibular canines. Patients were followed-up monthly, and activation of the appliance was done when necessary. Different complications that occurred during the therapy were reported and managed accordingly. Treatment was continued until an overcorrection to an edge to edge incisor relationship was achieved, and then the appliance was removed. The patients then continued their course of orthodontic treatment.

## RESULTS

Baseline characteristics for the involved subjects are presented in Table 1. The duration of the FFRD phase was 5.82 ± 1.15 months. FFRD was successful in correction of the Class II incisor and molar relationship in all subjects; where an edge to edge relationship was achieved. Cephalometric and clinical results are to be published in a separate text.

**Table 1:** Baseline characteristics of the included sample.

Age	12.4 ± 1.98
Overjet	6.17 ± 2.09
SNA	82.1 ± 2.33
SNB	74.9 ± 2.15
ANB	7.3 ± 1.88
MP/SN	35.7 ± 6.1
U1/PP	116 ± 4.53
L1/MP	97.8 ± 6.1

### The encountered complications

Data of any clinical complications that occurred during treatment were collected from the patients’ clinical records, which included pictures that were taken at the incidence of any complication. For the sake of simplification, these complications were summarized and subdivided into categories as presented in Table 2. The main categories were the complications related to problems in the FFRD and/or the fixed appliance; including breakage, separation of parts, spring fatigue and sheared molar tubes. The second category was concerned with the complications that were demonstrated in the patients’ teeth and/or soft tissues; including swelling, rotated and/or intruded teeth and canting in the occlusal plane.

**Table 2:** The complications encountered during the FFRD therapy of the included patients.

Category	Complication	No of occurrence	Percentage of occurrence
A) Complications shown on the appliance	Breakage of FFRD (Fig 1)	2/58	3.4%
	Fatigue of FFRD springs (Fig 2)	4/58	6.9%
	Separation of parts “patients who were not able to reassemble the parts”	5/58	8.6%
	Shearing off the pre-welded extraoral tubes from the upper first molar bands (Fig 3)	12/58	20.9%
	Total number of events	23	
B) Complications demonstrated on the patients’ teeth, intraoral and/or extraoral tissues	Extraoral swelling	3/58	5.2%
	Rotated lower canine(s)	30/58	51.7%
	Squeezing of the rotated lower canine(s) out of the arch (Fig 5A)	5/58	8.6%
	Increased upper molar intrusion and encroachment of the TPA on the palatal mucosa (Fig 6, 7)	4/58	6.9%
	Canting of the occlusal plane and a lateral open bite. (Fig 8A, B and C)	1/58	1.7%
	Development of posterior cross bite	15/58	25.9%
	Total number of events	58	
Total number of complications that occurred in the sample		n = 81	
Mean number of complications per patient “in the whole sample”		n = 1.40	
Mean number of complications per patient “in the patients who showed complications only from the sample (36 patients)”		n = 2.25	

The current study reported twenty-two patients to have a complications-free treatment. In other words, 36/58 patients experienced various complications during the appliance therapy, with an incidence of 62%. This percentage is similar to the one reported by Phuong et al.^12^ in their systematic review. The mean number of complications per patient was 1.4 when calculated over the whole sample. When the number of complications per patient was calculated over those who presented with complications, it increased to 2.25. Previous studies reported a range of 0.42 to 4.29 events per patient.^12^

## DISCUSSION

Reports on the complications of Herbst appliance and its variations are numerous in the literature.^13^ ^-^ ^16^ All previous studies evaluating the treatment side effects of FFRD did not include clinical findings, but were based on patients’ responses to pre-set questionnaires.^8^ ^-^ ^11^ Unlikely, this clinical report showed the other perspective, that is, the clinician’s observations. Assessment questionnaires are important to report patient acceptance and predict the expected level of compliance. However, they might have some disadvantages, for example, there is no way to tell how truthful a respondent is being, and there is also no way of assessment of the level of comprehension of the respondent to the questions. Moreover, subjectivity is always present because of the difference in perception between different respondents, and finally a risk of bias may exist when the researcher develops the questionnaire.^20^ Clinicians’ reports of complications are thus important to augment findings of different questionnaires of patients’ acceptance to various appliances. 

One of the factors that were accounted for in the current study was the level of clinician experience with the appliance. The cases of this clinical report were treated in a university setting and by the same clinician, who had been using the appliance three years before commencing the trial. It is believed that the level of operator experience with the appliance can affect the treatment progress, the incidence of complications and the overall patient experience. Lack of experience can account for multiple appliance breakages, failures and other complications. Bowman et al.,^8^ who reported on the patient experience with FFRD, mentioned that the doctor experience level could be considered a confounding factor in their study. The formerly mentioned study^8^ included a university clinic and private practice practitioners with varied levels of experience, which could have accounted for their higher reported breakage rates of the appliance. They recommended that further investigations should control the operator experience on examining the performance of the FFRD.

### A) Complications in the appliance

**Figure 1:**
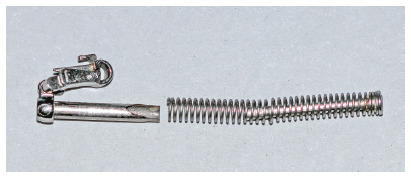
A broken FFRD spring.

FFRD breakage was encountered in this report in only two subjects. The first breakage was at the clip module of the spring, while the second was at the spring itself (Fig 1). Both appliances were replaced with new ones. FFRD breakage was reported to be higher in previous studies^6^ ^,^ ^8^; however, no data were given regarding whether the appliance itself or the orthodontic bands and/or wires were the broken parts. This could be an indication that FFRD might have less breakage rates when compared to the acrylic splint Herbst.^14^ ^,^ ^16^ However, it should be kept in mind that the reported treatment duration of the Herbst appliance ranged from 8 to 12 months,^21^ which is more than that for the FFRD. This longer duration can be the reason for the increased breakage incidence of the Herbst, as compared with the FFRD. 

**Figure 2:**
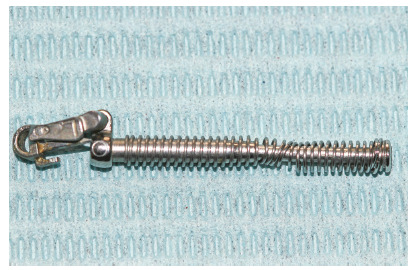
Fatigued spring of FFRD.

Although named FFRD, fatigue of the spring did occur unilaterally in four subjects of this report. The spring coils were uneven, and its springiness was markedly affected (Fig 2). Such an occurrence could be a result of over-activation of the appliance, which is a clinician-related problem. 

One of the main advantages of the FFRD is that the patients can reassemble the parts through wide mouth opening with compression of the spring to embrace the push rod. Only five patients were unable to reassemble the appliances’ parts on their own and they were educated to manage such an incident.

**Figure 3:**
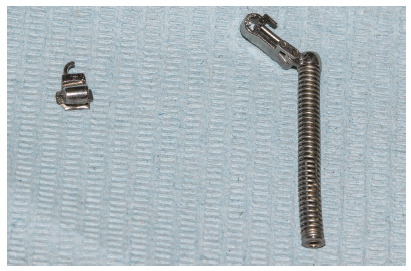
Sheared off pre-welded extraoral tube from a maxillary first molar band.

Shearing off the pre-welded extraoral tubes from the upper first molar bands was a common complication in the current report (Fig 3). It was accompanied with breakage of the molar bands in three subjects. This breakage was previously reported by Ross et al.^22^ to occur in one out of 17 FFRDs. Management of such an occurrence was by removal of FFRD, the TPA and the maxillary archwire, followed by construction of a new TPA with new molar bands. After cementation of the new TPA, FFRD was re-inserted and treatment was resumed. 

### B) Complications demonstrated on the patients’ teeth, intraoral and/or extraoral tissues

**Figure 4:**
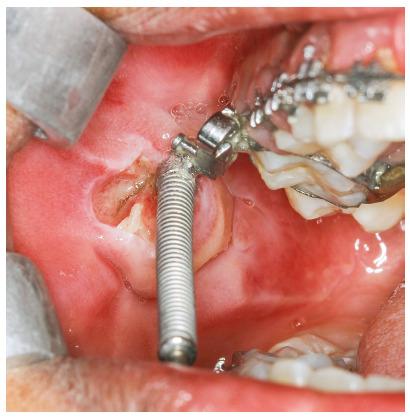
Intraoral view of ulcers related to the FFRD.

Extraoral swelling, that was coupled with poor oral hygiene and cheek ulceration (Fig 4), was one of the severe complications of the appliance. Cheek irritation was in accordance with the previous studies evaluating the patients’ experience with FFRD,^8^ ^,^ ^10^ but the extraoral swelling was not previously reported. Appliances were removed, and the patients were referred for periodontal and surgical consultation. Recurrence of the condition did not occur, which is in accordance with previous reports that the discomfort associated with FFRD usually diminished with time.^9^

**Figure 5:**
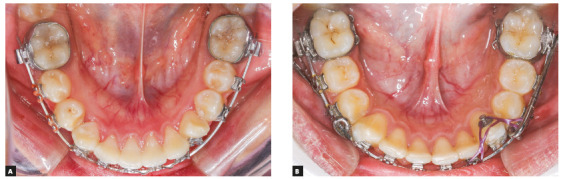
A) Severe mesio-lingual rotation of the mandibular left canine. B) Procedures done for re-alignment of the rotated mandibular canine.

Rotation of the lower canines is a common occurrence with FFRD therapy due to the continuous forces exerted by the push rods.^23^ It occurred in 30 cases in the current report. The severe rotation of the canine could be due to the gradual looseness of the canine ligation leading to mesial-in rotation caused by the continuous mesial force application to the canine bracket level buccal to its center of resistance. However, severe rotation that led to squeezing of the mandibular canine(s) out of the arch occurred unilaterally in five out of the 58 treated patients. Mesio-lingual canine rotation was managed by removal of the appliance and attempting to bring the canine back into the arch using elastic chains attached to a button bonded on the tooth lingual surface, pulling it towards the same rigid archwire (Fig 5B). After re-aligning the lower canines, treatment was resumed with re-insertion of the appliance.

**Figure 6:**
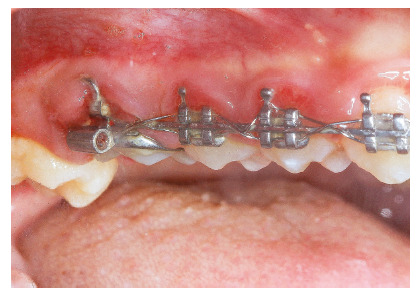
A vertical step between the intruded maxillary buccal segment and the unengaged second molar.

**Figure 7:**
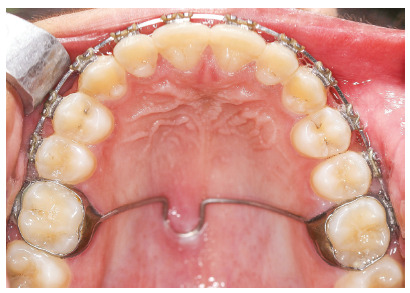
Encroachment of the TPA on the palatal mucosa.

Intrusion of upper first molars was reported as one of the treatment effects with FFRD,^24^ ^,^ ^25^ and occurred in all the patients in this report. However, a considerable step between the upper first and second molars was evident in four of our patients (Fig 6) and a lateral open bite was developed accordingly. One reason for this could be that the second molars were not bonded and not leveled with the maxillary wires. The exaggerated intrusion also led to encroachment of the TPA on the palatal mucosa causing its inflammation (Fig 7).

One of the findings that require further investigation is the difference in the amount of intrusion in different patients. It is believed that the FFRD delivers disto-gingival forces towards the maxillary molars. Upon resolution of these forces, the vertical and horizontal components are present and are inversely proportional to each other. As the horizontal component was reduced, the vertical component of force could be exaggerated. The horizontal component is related to the sagittal distance between the maxillary first molars and the mandibular canines (the length of the appliance). 

**Figure 8:**
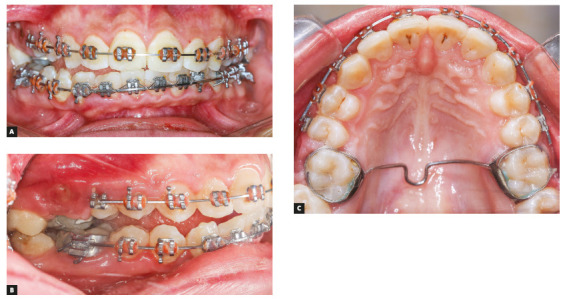
A) Canted mandibular occlusal plane. B) Lateral open bite associated with the canted occlusal plane. C) Occlusal view showing the broken maxillary first molar bands.

Missing appointments is a major cause of detrimental side effects of the orthodontic appliances.^26^ One of the patients included in this report presented with a combination of complications including canting of the lower arch, a lateral open bite on one side, a missing FFRD spring on the opposite side and broken maxillary molar bands on both sides (Fig 8). This patient did not show for three months in a row before she presented with this clinical picture. 

The occurrence of posterior crossbite during functional appliance therapy was previously reported in the literature because a wider posterior portion of the mandibular arch articulates in a forward position, with a narrower portion of the maxillary arch. Subsequently, it was advised to incorporate expansion screws in removable functional appliances.^27^ This side effect was managed by expansion of the TPA after the end of the FFRD phase, together with coordination of the archwires.

To our knowledge, this is considered the first clinical report to document the clinical complications faced during the FFRD therapy and how they were managed. Consequently, several preventive measures are presented to avoid such complications:

a) Proper patient education is mandatory before the start of the FFRD therapy. This should include instructions regarding a strict oral hygiene protocol, limitation of excessive lateral movements and wide mouth opening, to avoid separation of the appliance parts.
b) Avoid over-activation of the appliance. This could help to avoid breakage of the appliance and/or the molar bands and, thus, reduce the number of emergency appointments.
c) Proper ligation of the mandibular canines is required to avoid their excessive rotation and squeezing out of the arch. Other measures can help reduce this complication including the use of elastomeric ligatures with a bite guard (3M Unitek, Monrovia, CA, USA) and/or rotational wedges.^23^
d) Proper selection of the size of the FFRD is needed to avoid the excessive vertical component of force, which results in exaggerated molar intrusion.
e) Second molars need to be included and levelled in the maxillary arch before the start of FFRD therapy. This can help avoid the excessive first molar intrusion during the FFRD stage.
f) The TPA is to be fabricated with 1-2 mm relief from the palatal mucosa. Slight expansion of the TPA could be helpful to avoid crossbites.
g) Cheek irritation that is caused by FFRD should be addressed, since it is a commonly reported event. Recently, spring caps have been introduced to cover the anterior and/or the posterior end of the spring, which may be the reason of cheek and/or lower lip irritation.

## LIMITATIONS

The recruited sample included only females, which could have limited the generalizability of the study. Further studies are needed to compare between the complications induced by different appliances while recruiting bigger sample sizes.

## CONCLUSIONS

Different complications were encountered during the FFRD appliance therapy and were reported hereby. This report could help to undertake preventive measures for avoiding the occurrence of such incidences.
